# Emergence of a Hypervirulent Tigecycline-Resistant *Klebsiella pneumoniae* Strain Co-producing *bla*_NDM–1_ and *bla*_KPC–2_ With an Uncommon Sequence Type ST464 in Southwestern China

**DOI:** 10.3389/fmicb.2022.868705

**Published:** 2022-04-29

**Authors:** Jingchen Hao, Bangqin Zhang, Jiamin Deng, Yueshuai Wei, Xue Xiao, Jinbo Liu

**Affiliations:** ^1^Department of Laboratory Medicine, The Affiliated Hospital of Southwest Medical University, Luzhou, China; ^2^Department of Respiratory and Critical Care Medicine, The Affiliated Hospital of Southwest Medical University, Luzhou, China; ^3^Department of Laboratory Medicine, Southwest Medical University, Luzhou, China

**Keywords:** *Klebsiella pneumoniae*, NDM-1, KPC-2, tigecycline resistance, hypervirulent

## Abstract

Emergence of *bla*_NDM–1_ and *bla*_KPC–2_ co-producing *Klebsiella pneumoniae* strains is currently attracting widespread attention, but little information is available about their tigecycline resistance, virulence, and prevalence in Southwest China. In July 2021, an extensively drug-resistant *K. pneumoniae* strain AHSWKP25 whose genome contained both *bla*_NDM–1_ and *bla*_KPC–2_ genes was isolated from the blood of a patient with the malignant hematological disease in Luzhou, China. We investigated the resistance profiles of AHSWKP25 using microbroth dilution, agar dilution, modified carbapenemase inactivation (mCIM), and EDTA-modified carbapenemase inactivation methods (eCIM). The virulence of AHSWKP25 was assessed through string tests, serum killing assays, and a *Galleria mellonella* larval infection model. Conjugation and plasmid stability experiments were conducted to determine the horizontal transfer capacity of plasmids. And efflux pump phenotype test and real-time quantitative reverse transcription-PCR (RT-PCR) were used to determine its efflux pump activity. Sequencing of AHSWKP25 determined that AHSWKP25 belonged to ST464, which is resistant to antibiotics such as carbapenems, tetracycline, fluoroquinolones, tigecycline, and fosfomycin. The efflux pump phenotype tests and RT-PCR results demonstrated that efflux pumps were overexpressed in the AHSWKP25, which promoted the tigecycline resistance of the bacteria. AHSWKP25 also showed hypervirulence and serum resistance *in vitro* model. AHSWKP25 carried several different plasmids that contained *bla*_NDM–1_, *bla*_KPC–2,_ and mutated *tet(A)* genes. Sequence alignment revealed that the plasmids carrying *bla*_NDM–1_ and *bla*_KPC–2_ underwent recombination and insertion events, respectively. We demonstrated that an X3 plasmid carrying *bla*_NDM–1_ was transferred from pSW25NDM1 to *E. coli* J53. We also identified missense mutations in the *ramR*, *rcsA*, *lon*, and *csrD* genes of AHSWKP25. Our results highlighted the potential of *bla*_NDM–1_ and *bla*_KPC–2_ co-producing *K. pneumoniae* strains to further develop antimicrobial resistance and hypervirulent phenotypes, but measures should be taken to closely monitor and control the spread of superbugs with multidrug-resistant phenotypes and hypervirulence.

## Introduction

*Klebsiella pneumoniae* is a common Gram-negative bacteria without spores or flagella that belongs to the *Bacillus* genus. In clinical infections involving *K. pneumoniae*, the classical (cKp) and hypervirulent (hvKp) strains are the most common, and the hvkp strains are considerably more dangerous because they can infect multiple organs, leading to high mortality rates from multiorgan failure. The pathogenicity of hvKp is usually attributed to multiple virulence factors, one of the most representative of which is the capsular antigen, which not only enhances virulence but also allows the bacterium to evade the host’s immune response (Podschun and Ullmann, 1998). There are six main types of the capsular antigen that enable the invasion of the species: K1, K2, K5, K54, K20, and K57. These antigens together with other virulence factors form the hypervirulence phenotype of *K. pneumoniae* (Turton et al., 2010; Shon et al., 2013). K1 and K2 are the most well-described capsular serotypes and are often detected in the *K. pneumoniae* multilocus sequence types ST23 and ST65 (Liao et al., 2014). Virulence plasmids are a significant marker of these hvkp strains (Shon et al., 2013). The *rmpA* gene product regulates the synthesis of capsular polysaccharides, conferring a hypermucoviscous phenotype on these pathogens (Cheng et al., 2010). A recent study revealed that, in addition to *rmpA*, the presence of *rmpC* and *rmpD* genes at the *rmp* locus also contributed to the formation of the hypermucoviscous *K. pneumoniae* phenotype (Walker et al., 2020). However, not all hypervirulent strains have a mucinous phenotype or carry the *rmpA*/*rmpA2* or *iucA* genes (Yan et al., 2021). One study indicated that mutations in the genes (wzi, wza, wzc, *rcsAB*, and *lon*) that encode the enzymes and regulators responsible for capsule production contributed to aberrant capsule production that promoted the pathogenicity and antiserum phagocytosis of *K. pneumoniae* strains. And these mutants producing hypercapsule are often associated with bloodstream infection (Ernst et al., 2020). Therefore, more studies are required to better understand the hvKp strains.

The resistance status of hvKp is not promising. Like cKp, hvKp can acquire resistance to antimicrobial agents with the acquisition of mobile elements carbapenem-resistant-hvKp is a typical example of a stain that can acquire resistance to various antimicrobial agents by obtaining exogenous plasmids carrying antimicrobial resistance genes (Yao et al., 2015). cKp can also develop into hypervirulent strains by acquiring virulence plasmids. The high pathogenicity and antimicrobial resistance of these pathogens translate to high treatment costs and poor prognoses (Shankar et al., 2018). Colistin and tigecycline represent the last line of defense against carbapenem-resistant Enterobacteriaceae bacteria, especially metallo-beta-lactamase-producing strains (Maltezou, 2009). The horizontal transfer of *mcr*, *tet(A)*, and *tet(X)* allowed *K. pneumoniae* to rapidly acquire resistance to colistin and tigecycline. Also, disruptions in chromosomal genes (*mgrB*, *pmrAB*, and *phoPQ*), as well as resistance nodulation cell division (RND)-type efflux transporters and their regulators (*marRA*, *ramRA*, and *rarA*), are prominently responsible for conferring resistance of these microorganisms to antimicrobial agents (Osei Sekyere et al., 2016; Poirel et al., 2017). Recently, one study reported the emergence of *bla*_NDM–1_ and *bla*_KPC–2_ co-producing *K. pneumoniae* strains that could have both high-level carbapenem resistance and hypervirulent phenotypes (Liu et al., 2019; Gao et al., 2020). However, little information is available on the virulence characteristics, or tigecycline resistance of the *bla*_NDM–1_ and *bla*_KPC–2_ co-producing *K. pneumoniae* strains. To monitor these bacteria and prevent the development of further antimicrobial resistance, more information on these strains is required. In this study, we isolated an extensively drug-resistant *K. pneumoniae* isolate (AHSWKP25) from the blood of a patient with a bloodstream infection. AHSWKP25 was found to be an isolate harboring *bla*_NDM–1_, *bla*_KPC–2,_ and *tet(A)* with an uncommon sequence type and was resistant to tigecycline and fosfomycin. In this study, we examined the virulence, genetic characteristics, and resistance mechanisms of AHSWKP25.

## Materials and Methods

### Source of the Isolate

In July 2021, AHSWKP25 was isolated from the blood of a patient diagnosed with acute myeloid leukemia with a bloodstream infection at the Affiliated Hospital of Southwest Medical University (Luzhou, China). The patient had previously been tread with ceftazidime, meropenem, moxifloxacin, tigecycline, and voriconazole for fungal infection and repeated fever. The clinical microbiology laboratory identified AHSWKP25 as *K. pneumonia* that was resistant to carbapenems, tigecycline, fluoroquinolones, and other common antimicrobial agents. The patient ultimately died of multiorgan failure. To better understand the antimicrobial resistance mechanisms and virulence characteristics of AHSWKP25, we undertook a series of experiments described below.

### Antimicrobial Susceptibility Testing, Efflux Pump Phenotype Test, and DNA Amplification

The antimicrobial minimum inhibitory concentrations (MICs) of ceftazidime, cefepime, gentamicin, amikacin, chloramphenicol, ciprofloxacin, levofloxacin, tetracycline, polymyxin B, ceftazidime/avibactam, fosfomycin (agar dilution method), and tigecycline were determined according to the (Clinical and Laboratory Standards Institute (CLSI), 2020) standards. The MIC breakpoints of imipenem, ertapenem, aztreonam, levofloxacin, nitrofurantoin, and trimethoprim-sulfamethoxazole were determined using the MicroScan Walk Away System (Siemens, Germany). The breakpoint of tigecycline was interpreted according to the guidelines of the United States Food and Drug Administration (FDA^1^) on Antimicrobial Susceptibility Testing. The modified carbapenemase inactivation (mCIM) and EDTA-modified carbapenemase inactivation methods (eCIM) were also used following the CLSI 2020 standards.

To evaluate the efflux pump activity of AHSWKP25, we first measured the changes in the MICs of several antimicrobial agents in the presence of 1-(1-Naphthylmethyl)-piperazine (NMP, 100 mg/L). A fourfold or greater reduction in MIC was considered an indicator of overexpression in the efflux pumps (Schumacher et al., 2006). Polymerase chain reaction (PCR) was performed to detect whether the isolate carried the *bla*_KPC_ or *bla*_NDM_ genes, and the primers used are listed in Supplementary Table 1.

### String Test, Serum Killing Assay, and *Galleria mellonella* Infection Model

The hypermucoviscous phenotype was determined using a string test. A single purified colony on a blood agar plate incubated at 37°C for 18-24 hours was picked with an inoculation loop for string, and the string test was considered positive if the length of the viscous string was longer than 5 mm (Yan et al., 2021). A serum killing assay was performed as described previously. In brief, 25 μL of the bacterial suspension (concentration of ∼1 × 10^6^ CFU/mL) was added to 75 μL of healthy human serum for co-culture. After 0, 1, 2, and 3 h, the plates were inoculated with the mixture and the numbers of viable bacteria were calculated. The strains were classified as “highly sensitive,” “moderately sensitive,” or “resistant” to serum, depending on the results (Liu et al., 2017).

The pathogenicity of AHSWKP25 was assessed using a *G. mellonella* larvae infection model as previously described (Kim et al., 2021). A total of 15 healthy vigorous larvae were inoculated with the bacterial suspension of AHSWKP25 isolate at a dose equivalent to 10^6^CFU. When the larvae were inactive and black, they were considered dead. The numbers of larval deaths were recorded every 12 h. *K. pneumoniae* NTUH-K2044 and ATCC 700603 were used as the hypervirulent control and negative control, respectively. The bacterial suspension was serially diluted and injected into *G. mellonella* larvae for 3 days of incubation, and the lethal dose 50 (LD_50_) was calculated using the probit model (Shi et al., 2018).

### Whole Genome Sequencing, Identification of Mutant *rcsAB*, *lon*, *csrD*, and *pal*, Phylogenetic Reconstruction

Luria-Bertani (LB) broth was inoculated with the selected purified *Klebsiella* colonies, and the bacteria were cultured to log phase. Extraction of bacterial genomic DNA using a magnetic bead-based kit (Qiagen, Germany). The extracted bacterial DNA was purified and sequenced using the Illumina NovaSeq PE150 and Oxford nanopore platforms. The bacterial genome was assembled *de novo* using Canu (v 1.7). Prokka (v 1.10) was used to predict and annotate the coding genes, tRNAs, and rRNAs in the assembled genome. The plasmid replicon types, acquired resistance genes, sequence types, and virulence genes were determined using the online services of the Center for Genomic Epidemiology^2^ and VFDB (virulence factor database^3^). IS finder^4^ was used to identify the insertion sequences. The genomes were compared using the Basic Local Alignment Search Tool (BLAST^5^), BRIG v0.95, Mauve^6^, and the OAT software (Lee I. et al., 2016).

We used *K. pneumoniae* UCI 38 (accession number: JCMB01) as a reference strain to identify point mutations in the *rcsAB* and *lon* (Lon protease) genes of AHSWKP25 to evaluate whether AHSWKP25 was associated with hypercapsule production (Ernst et al., 2020). The *rscD* and *pal* mutants were identified using *K. pneumoniae* ATCC13883 (accession number: JOOW01) as the reference genome.

A goeBURST full MST analysis based on multilocus sequence typing data was performed by the Phyloviz 1.1a software to infer the phylogeny of the sequence types^7^. The Orthologous average nucleotide identity (OrthoANI) was calculated by comparing homologous genes between the genomes to construct a phylogenetic tree (Lee I. et al., 2016), which was visualized by iTOL^8^. The accession numbers of the genomes/sequences obtained from the NCBI database were listed in Supplementary Table 2.

### Measurement of Efflux Pump Transcription Levels Using Quantitative Reverse Transcription-PCR

To evaluate the role of chromosomal point mutations in the antimicrobial resistance of AHSWKP25, we used RT-PCR to measure the transcriptional levels of efflux pump-encoding genes (*acrA*, *acrB*, *marA*, *marR*, *rarA*, and *ramA*) involved in conferring tigecycline resistance. In brief, single purified colonies of AHSWKP25 were inoculated into 5 mL of LB broth and the bacteria were grown to log phase. Their RNA was extracted according to the recommendations of the reagent’s manufacturer (Magen, China). The RT-PCR primers used in the present study are listed in Supplementary Table 1. The *rpoB gene* was used as the internal reference, and the tigecycline-susceptible *K. pneumoniae* strain NTUH-K2044 was used as the control strain. All experiments were performed in triplicate independently, with three biological replicates per experiment. The gene expression results were compared to the expression levels of *rpoB* to calculate the relative expression of the target genes (2^–ΔΔCt^ method).

### Conjugation Experiments and Plasmid Stability

Sodium azide-resistant *Escherichia coli* J53 was used as the recipient and AHSWKP25 as the donor in the conjugation experiments. The McFarland (McF) standard turbidity of the bacterial suspension was adjusted to 0.5 McF. The recipient bacteria (200 μL) and donor bacteria (400 μL) were inoculated into LB broth (800 μL) (Xiang et al., 2020). After 16–18 h of culturing at 35°C, MH plates containing 180 μg/mL sodium azide and antimicrobial agents [4 μg/mL meropenem; 4 μg/mL meropenem + 5 mM EDTA (final concentration); 0.25 μg/mL ciprofloxacin] were inoculated with 100 μL of the culture solution to screen for transconjugants. The transferred genes in the transconjugants were confirmed by PCR using the primers listed in Supplementary Table 1.

The stability of the self-transferred plasmids in the positive transconjugants was calculated using the plate count method, as described previously with some modifications (Nang et al., 2018). Briefly, antibiotic-free LB broth was inoculated with the positive transconjugants at a ratio of 1,000:1, and the bacteria were passaged continuously for 10 days. The mixed culture was sampled every day by inoculating aliquots (100 μL) of the mixed culture into meropenem-containing (4 μg/mL), antibiotic-free LB agar plates. The plates were incubated for 24 h to calculate the plasmid retention rate.

## Results

### Antimicrobial Susceptibility and General Characteristics of AHSWKP25

The antimicrobial susceptibility tests indicated that AHSWKP25 was resistant to common antibacterial agents, such as meropenem, tetracycline, ciprofloxacin, levofloxacin, and gentamicin. Notably, AHSWKP25 was also resistant to fosfomycin, tigecycline (16 μg/mL), and ceftazidime-avibactam, as shown in Table 1. The mCIM and eCIM results suggested that AHSWKP25 carries serine carbapenems. Subsequently, PCR and Sanger sequencing confirmed that AHSWKP25 was a *bla*_NDM–1_ and *bla*_KPC–2_ co-producing *K. pneumonia* isolate.

**TABLE 1:** Antibiotic susceptibility tests of AHSWKP25 and its conjugants, and efflux pump phenotype test of AHSWKP25.

Antibiotics			MIC(μg/ml)	
	
	AHSWKP25	AHSWKP25 + NMP (100 μg/mL)	J53	J53 + pSW25NDM1
Ceftazidime	>128 (R)	>128 (R)	**/**	**/**
Meropenem	64 (R)	64 (R)	0.5 (S)	8 (R)
Tetracycline	>128 (R)	128 (R)	1	1
Ciprofloxacin	8 (R)	1 (R)	0.0625 (S)	0.0625 (S)
Gentamicin	32 (R)	16 (R)	0.5 (S)	0.5 (S)
Chloramphenicol	>128 (R)	64(R)	**/**	**/**
Polymyxin B	2 (I)	2 (I)	1(I)	1(I)
Tigecycline	16 (R)	1 (S)	0.125 (S)	0.125 (S)
Cefepime	>128 (R)	**/**	1	128 (R)
Aztreonam	>16 (R)	**/**	**/**	**/**
Imipenem	>8 (R)	**/**	**/**	**/**
Levofloxacin	>4 (R)	**/**	**/**	**/**
Ceftazidime/Avibactam	>32/4 (R)	**/**	**/**	**/**
Trimethoprim/Sulfamethoxazole	>4/76 (R)	**/**	**/**	**/**
Fosfomycin	>256 (R)	**/**	**/**	**/**

**FIGURE 1:**
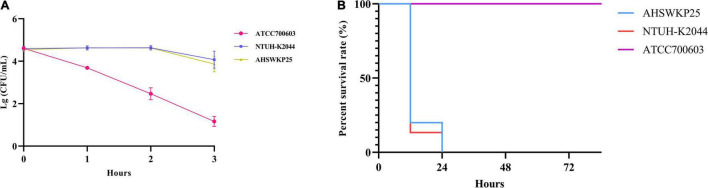
Serum resistance and pathogenicity of AHSWKP25.**(A)** The survival rate of AHSWKP25, ATCC700603, and NTUH-K2044 in healthy human serum over 3 h. The data are expressed as the mean ± SEM (standard error of the mean). **(B)** The survival rate of *G. mellonella* larvae (15 biological replicates) inoculated with AHSWKP25, NTUH-K2044, and ATCC700603, respectively. The experiments were repeated in triplicate independently.

The string test of AHSWKP25 was negative, as the mucoid string length was < 5 mm. AHSWKP25 was highly pathogenic in the *G. mellonella* infection model. Of the 15 *G. mellonella* larvae that were inoculated with 10^6^ CFU of AHSWKP25, 15 died within 24 h, corresponding to a mortality rate of 100%, which was similar to the rate for the hypervirulent control strain NTUH-K2044 (*P* > 0.05) but different from that of *K. quasipneumoniae* ATCC700603 (negative control, *P* < 0.0001) (Figure 1B). The LD_50_ of AHSWKP25 was 4.44 ± 0.23 (log10 CFU), and the LD_50_ of the NTUH-K2044 was 4.21 ± 0.17 (log10 CFU) (*P* > 0.05, Supplementary Figure 1). Both NTUH-K2044 and AHSWKP25 showed resistance to the serum, whereas ATCC 700603 was highly sensitive to the serum (Figure 1A and Supplementary Table 3). To further investigate the virulence characteristics and resistance mechanisms of AHSWKP25, we sequenced its whole genome to identify mutations in the genes that regulate the resistance mechanisms.

### Sequence Characteristics of the AHSWKP25 Genome

**FIGURE 2:**
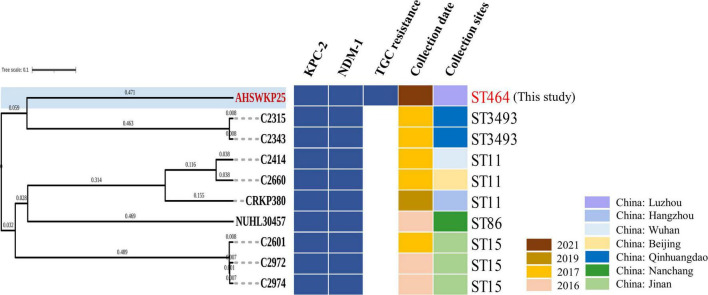
OrthoANI-based phylogenetic tree of AHSWKP25 and other 9 NDM-1 and KPC-2 co-producing *K. pneumoniae* strains previously isolated from other parts of China. The different colors, respectively represented the resistance genes, collection date, and sites, blanks represent strains that are susceptible to tigecycline.

The full length of the AHSWKP25 genome was 5,874,657 bp, and it consisted of a chromosomal backbone carrying *bla*_SHV–5_ and seven circular plasmids. Of these, the plasmid incompatibility (Inc) groups for pSW25NDM1, pSW25HRG, and pSW25tet(A) were X3, FIB, and FII/FIA, respectively. The *bla*_KPC–2_ and *bla*_NDM–1_ genes were located on plasmids pSW25KPC2 and pSW25NDM1, respectively. The AHSWKP25 genome was longer than *K. pneumoniae* MGH78578 (5,694,894 bp, GenBank accession number: CP000647-652) and ATCC13883 (5,545,784 bp, accession number: JOOW01). The capsular serotype of AHSWKP25 was identified as K53 based on the wzc locus, which was consistent with the serotype of *K. quasipneumoniae* ATCC700603 (GenBank accession number: CP014696.2). The wzi allele (wzi outer membrane protein of cluster) of AHSWKP25 was most similar to allele 534 but it differed by one base pair. Therefore, we uploaded the wzi allele of AHSWKP25 into a public database^9^ and assigned a novel profile to it defined as wzi 725. The *mrkABCDFHIJ*, *fimABCDEFGHIK*, *rcsAB*, *entABCDEFS*, and *iroE* genes were all identified on the chromosomal backbone of AHSWKP25, whereas the *iucA* and *rmpA*/*rmpA2* genes were not detected (Supplementary Table 4). AHSWKP25 shared an average nucleotide identity (ANI) of 98.97 and 98.96% with *K. pneumonia* MGH78578 and ATCC 13883 (Supplementary Figure 2), respectively. The multilocus sequence type of AHSWKP25 was ST464, which differed from ST4292 and ST2439 by only one allele, whereas ST464 differed from ST11 by five alleles (Supplementary Figure 3). The amino acid substitutions in *ramR*, *acrR*, and *ompK36*/*ompK37* genes of AHSWKP25, as shown in Table 2. Furthermore, we also identified Ser35Asn, Glu259Ala, and Gln110His missense mutations in the *rcsA*, *lon*, and *csrD* genes of AHSWKP25, respectively. However, no missense mutations were found in the *pal* gene. A phylogenetic tree generated from orthoANI data revealed that AHSWKP25 and the *K. pneumoniae* strains co-producing NDM-1 and KPC-2 previously isolated from other regions of China (Liu et al., 2019; Gao et al., 2020; Xu et al., 2020) had obvious regional and temporal differences and were significantly separated phylogenetically (Figure 2).

**TABLE 2:** Genomic characterization of AHSWKP25.

ID	Size (bp)	GC Ratio	Resistance genes^a^	Point mutations^a^	Incompatibility group^a^
Chromosome	5,244,699	0.5759	*bla*_SHV–5_, *fosA*	*ramR* (D152Y, K194*); *ompK36* (N49S, L59V, L191S, F207W, A217S, N218H, D224E, L228V, E232R, N304E); *ompK37* (I70M, I128M); *acrR* (P161R, G164A, F172S, R173G, L195V, F197I, K201M); *rcsA* (S35N)^b^; *lon* (E259A)^b^; *csrD* (Q110H)^c^	NA
pSW25KPC2	248,847	0.4661	*aadA16*, *qnrB4*, *aac(3)-IId*, *sul1*, *ARR-3*, Δ*bla*_TEM_, *dfrA27*, *bla*_KPC–2_, *qacE*Δ*1*, *merR*	NA	Unknown
pSW25HRG	140,379	0.4968	*pcoABCDERS*, *silBCEFGPRS*, *terABCDEWXYZ*	NA	FIB
pSW25tet_A	120,990	0.5334	*tet(A)*, *qnrS1*, *dfrA14*, *sul2*, *bla*_LAP–2_, *floR*	*tet(A)*^d^ (I5R, V55M, I75V, T84A, S201A, F202S, V203F)	FII/FIA
pSW25NDM1	59,349	0.4906	*bla*_SHV–12,_ *bla*_NDM–1_	NA	X3
pSW25H01	54,244	0.5176	NA	NA	Unknown
pSW25H02	3,482	0.5017	NA	NA	Unknown
pSW25H03	2,667	0.4578	NA	NA	Unknown

*NA, not applicable; ^a^Data was obtained from the online website (https://cge.cbs.dtu.dk/, https://bigsdb.pasteur.fr/), ^b^Reference sequence: K. pneumoniae UCI 38 (accession number: JCMB01), ^c^Reference sequence: K. pneumoniae ATCC 13883 (accession number: JOOW01), d: Reference sequence: tet (A) (GenBank accession number: X00006).*

### Sequence Analysis of Plasmids Carried by AHSWKP25

**FIGURE 3:**
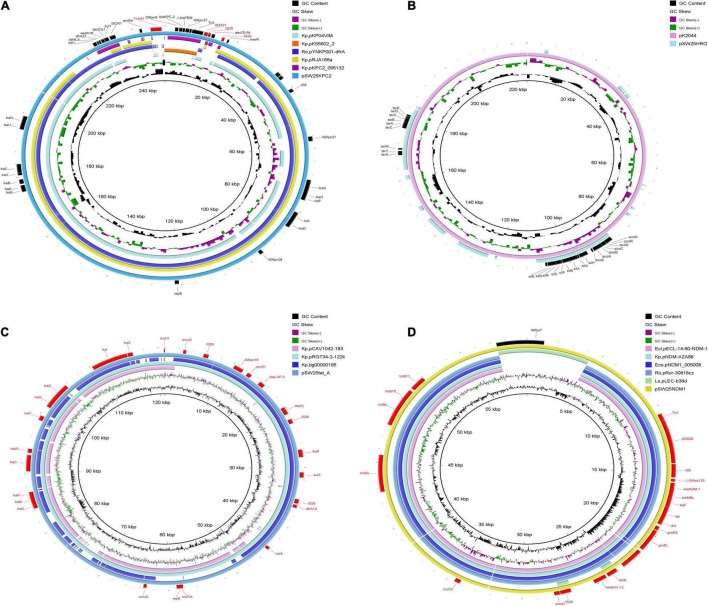
Comparative genomic circle of plasmids. **(A)** Circular comparison of pSW25KPC2 with other similar plasmids, pSW25KPC2 was used as a reference. **(B)** Circular comparison between pSW25HRG and virulence plasmid pK2044, pK2044 was used as a reference. **(C)** Circular comparison of pSW25tet_A with other similar plasmids, pSW25tet_A was used as a reference. **(D)** Circular comparison of pSW25NDM1 with other similar plasmids, pSW25NDM1 was used as a reference. The outermost circle annotates the genetic information, and different plasmids are assigned different colors. Blanks represent deleted regions compared to the reference plasmids. Kp: *K. pneumoniae*, Ro: *R. ornithinolytica*, Ecl: *E. cloacae*, Eco: *E. coli*, Ls: *Leclercia* sp.

**FIGURE 4:**
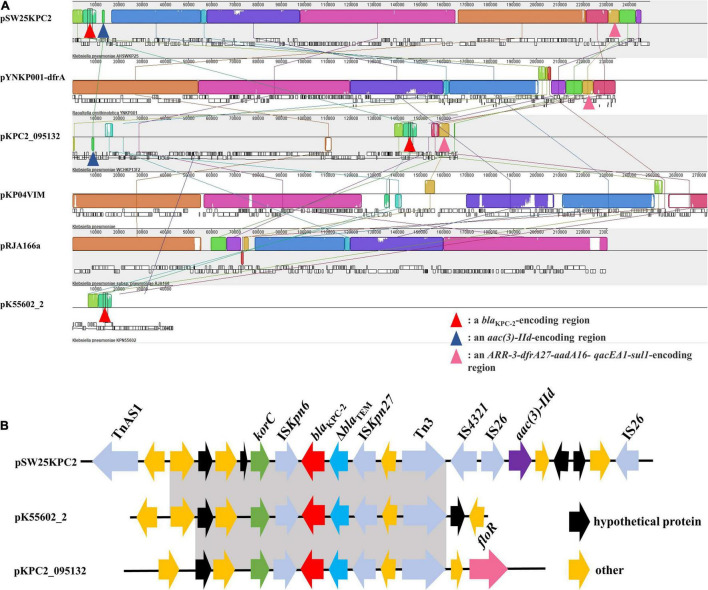
**(A)** Mauve alignment between pSW25KPC2 and other five plasmids. The grids of the same color represent collinear areas and are connected by lines. The blank area in the grid indicates that the sequences are incompletely aligned. **(B)** Comparison of genetic elements surrounding *bla*_KPC–2_ between pSW25KPC2 and plasmids carrying *bla*_KPC–2_. Gray areas represent homologous portions between sequences.

Among the seven circular plasmids carried by AHSWKP25, we focused on four of them, namely pSW25KPC2, pSW25HRG, pSW25tet_A, and pSW25NDM1. pSW25KPC2 was a circular plasmid with a length of 248,847 bp, but we failed to identify its incompatibility group with Plasmid Finder 2.1. This plasmid carried multiple resistance determinants, including *bla*_KPC–2_ (beta-lactam resistance), *aadA16* (aminoglycosides resistance), *aac(3)-IId* (aminoglycosides resistance), *sul1* (sulfamethoxazole resistance), *dfrA27* (trimethoprim resistance), *qnrB4* (quinolones resistance), *ARR-3* (rifampicin resistance), *merR* [Hg(II)-responsive transcriptional regulator] and *qacE*Δ*1* (quaternary ammonium compound resistance) (Figure 3A). We searched the National Center for Biotechnology Information (NCBI) database and found that pSW25KPC2 shared a 99.97% identity (with an 92% query coverage) compared to the plasmid pYNKP001-dfrA isolated from *R. ornithinolytica* (GenBank accession number: KY270853.1). Sequence alignment indicated that the backbone of pSW25KPC2 was highly similar to the plasmids pKP04VIM (*K. pneumoniae*), pYNKP001-dfrA (*R. ornithinolytica*), and pRJA166a (*K. pneumoniae*), but there were multiple Local Colinear Blocks (LCBs) that were inverted (Figures 3A, 4A). Notably, pKP04VIM, pYNKP001-dfrA (*K. pneumoniae*), and pRJA166a (*K. pneumoniae*) did not carry the *bla*_KPC–2_ gene. Further analysis revealed that the *bla*_KPC–2_ gene of pSW25KPC2 was located in an LCB of about 6 kb that was highly homologous with pK55602_2 (GenBank accession number: CP042976.1) and pKPC2_095132 (GenBank accession number: CP028389.3) and was highly collinear with no inversions or rearrangements. The mapping of this LCB to Figure 4B revealed that pSW25KPC2, pK55602_2, and pKPC2_095132 all contained a conserved region carrying *korC* (encoding transcriptional repressor protein), IS*Kpn6*, *bla*_KPC–2_, IS*Kpn27*, Tn*3*, and a truncated *bla*_TEM_. Transposase Tn*As1* (Tn*3*-like element) and IS*4321* were also present on the pSW25KPC2 plasmid flanking a region encoding *bla*_KPC–2_. Additionally, a region encoding *aac(3)-IId* was inverted in pSW25KPC2 compared to pKPC2_095132 (Figure 4A). The Tn*4401* transposon and its variants were not identified in pSW25KPC2. Lastly, pSW25KPC2 also contained a region connecting *ISCR1* and *intl1*, that contained *ARR*-3-*dfrA27*-*aadA16*- *qacE*Δ*1*-*sul1* genes (Figure 3A).

pSW25HRG was a FIB plasmid that did not carry any acquired antimicrobial resistance genes. There were several heavy metal resistance genes in the backbone of pSW25HRG: *pcoABCDERS* (copper resistance), *silBCEFGPRS* (silver resistance), and *terABCDEWXYZ* (tellurium resistance). Sequence alignments constructed with BLAST (see text footnote 5) revealed that most plasmid sequences similar to pSW25HRG have been isolated from *K. pneumoniae* strains. pSW25HRG shared a 99.93% identity (with an 80% query coverage) with a plasmid isolated from the *K. pneumoniae* strain FDAARGOS_1322 (GenBank accession number: CP070038.1) and a 99.90% identity (with an 83% query coverage) with a plasmid isolated from *K. pneumoniae* strain NICU_2_P7 (GenBank accession number: CP060050.1). Notably, pSW25HRG had a 23-kb homologous region compared to the well-known virulence plasmid pK2044 (GenBank accession number: NC_006625.1), wherein the above mentioned heavy-metal-resistance genes mainly occurred (Figure 3B).

The pSW25tet_A carried two replicon types, FII and FIA, and was 120,990 bp in length. Similar to pSW25KPC2, pSW25tet_A carried many genes encoding type IV conjugative transfer systems and multiple antimicrobial resistance determinants, including *tet(A)* (tetracycline resistance), *dfrA14* (trimethoprim resistance), *sul2* (sulfamethoxazole resistance), *qnrS1* (quinolones resistance), and *bla*_lAP–2_ (penicillins resistance). We identified that *tet(A)* in the pSW25tet_A was a type I mutant with a mutation profile of I5R, V55M, I75V, T84A, S201A, F202S, and V203F. This *tet(A)* mutant was previously demonstrated to be associated with reduced tigecycline susceptibility (Chiu et al., 2017). By searching the NCBI database, we found that pSW25tet_A was also similar to several other plasmids isolated from *K. pneumoniae* strains. Of these, pSW25tet_A shared a 99.97% identify (with a 100% query coverage) with a plasmid pRGT34-2-122k (GenBank accession number: CP075310.1) that was also isolated from *K. pneumoniae*. The homology between pSW25tet_A and pRGT34-2-122k was confirmed by comparing genomic circles (Figure 3C). pSW25tet_A shared only 28 and 29% coverage compared with pYUSHP2-2 (GenBank accession number: CP073773.1), a plasmid isolated from *E. hormaechei*, and pWP8-W19-CRE-01_3 (GenBank accession number: AP022271.1), a plasmid isolated from *R. ornithinolytica*, respectively.

Lastly, pSW25NDM1 was identified as an X3 plasmid carrying *bla*_NDM–1_ and *bla*_SHV–12_ genes. It was 59,349 bp in length and had a GC ratio of 49.06%. The genetic structure surrounding *bla*_NDM–1_ included IS*3000*, IS*5*, *ble*_*MBL*_, *trpF*, *tat*, *dct*, *groES* and *groEL*. pSW25NDM1 shared a 100% identity (with an 89% query coverage) with pECL-14-60-NDM-1 (GenBank accession number: MN061454.1), a plasmid isolated from *E. cloacae*. Sequence alignment revealed that pSW25NDM1 was very similar to plasmids isolated from *K. pneumoniae*, *E. coli*, *E. cloacae*, and *R. ornithinolytica* (Figure 3D). pSW25NDM1 contained an additional region of about 6 kb, which was not present in other plasmids carrying NDM-1, and it was highly homologous to a plasmid isolated from *Leclercia* sp. (GenBank accession number: CP026168.1) (Figure 3D). Further analysis showed that this region could be inserted by IS*Ro7* (marked black in Figure 3D). The *bla*_SHV–12_ was located in a 4-kb inserted region that could be inserted by IS*26* at both ends.

### Stable Dissemination and Expression of pSW25NDM1 Carrying *bla*_NDM–1_ Among Species

Among the three plasmids carrying the acquired antimicrobial resistance genes mentioned above, we only demonstrated the horizontal transferability of pSW25NDM1 in conjugation experiments. The pSW25NDM1 can be transferred to *E. coli* J53 at a transfer frequency of about 2.03 × 10^–3^. Recipient strain *E. coli* J53 showed significantly reduced susceptibility to meropenem and cefepime after the acquisition of plasmid pSW25NDM1 (Table 1). *E. coli* J53 carrying pSW25NDM1 displayed high plasmid stability during its continuous passage in an antibiotic-free environment for 10 days, and more than 95% of the recipient strains cells still retained pSW25NDM1 on day 10 (Supplementary Figure 4).

### Efflux Pump Phenotype Test and RT-PCR

**FIGURE 5:**
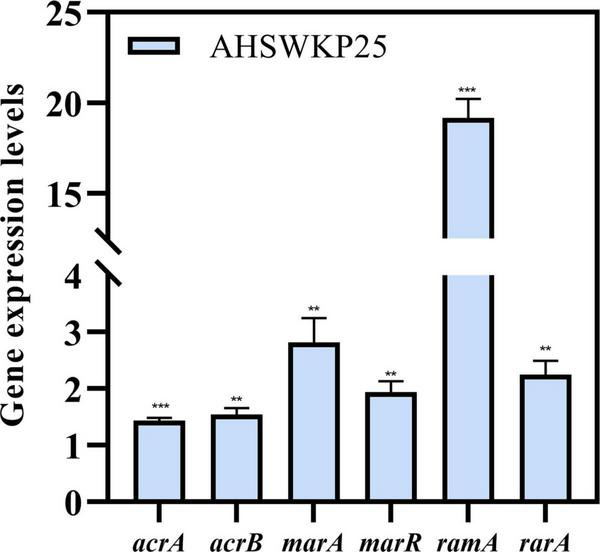
Relative expression levels of *acrA*, *acrB*, *marA*, *marR*, *ramA*, and *rarA* genes in AHSWKP25. Gene expression levels were normalized using the *rpoB* (RNA polymerase) gene. The experiment was repeated in triplicate independently, and data are expressed as the mean ± SD (standard deviation). **P < 0.01; ***P < 0.001 by Student’s *t*-test.

The MICs of ceftazidime and meropenem for AHSWKP25 did not change significantly after the addition of NMP (100 μg/mL**).** However, there were ≥ 4-fold reductions in the MICs of ciprofloxacin and chloramphenicol in the presence of NMP (100 μg/mL). And NMP restored the susceptibility of AHSWKP25 to tigecycline (Table 1). Furthermore, the RT-PCR results indicated that the expressions of *acrA* and *acrB* genes were up-regulated by 1.4-fold (1.43 ± 0.05) and 1.5-fold (1.54 ± 0.12), respectively, in AHSWKP25 compared to that in NTUH-K2044. The expressions of *rarA*, *marA*, and *marR* were up-regulated approximately twofold in AHSWKP25 (2.25 ± 0.24, 2.82 ± 0.43, 1.94 ± 0.19, respectively), while *ramA* was up-regulated 19-fold (19.18 ± 1.04) (Figure 5).

## Discussion

In this study, we reported on a *bla*_NDM–1_ and *bla*_KPC–2_ co-producing *K. pneumoniae* isolate (AHSWKP25) that was isolated in Southwestern China. To the best of our knowledge, this is the first report of a tigecycline resistant *K. pneumoniae* wzi 725 strain co-producing *bla*_NDM–1_ and *bla*_KPC–2_ in China, and its resistance profiles meet the definition of extensively drug-resistant (Magiorakos et al., 2012). In particular, AHSWKP25 belonged to an uncommon sequence type ST464, that was previously identified in neonatal patients in a few countries and for which little formation is available (Rakovitsky et al., 2020; Sands et al., 2021). We searched the database and found here was a one-allele difference between ST464 and ST2439 and two-allele differences between ST464 and ST5620, respectively. ST464 is markedly different from the sequence types of other *bla*_NDM–1_ and *bla*_KPC–2_ co-producing *K. pneumoniae* strains that have been reported in previous studies, suggesting that the combination of *bla*_KPC–2_ and *bla*_NDM–1_ not only appeared in several common sequence types (Figure 2 and Supplementary Figure 3). Therefore, continuous monitoring of such bacteria is still necessary. To date, we have not isolated any other *K. pneumoniae* strains with the same resistance pattern and sequence type as AHSWKP25 from other patients and settings in the local hospital. However, the patient was dead, and we were unable to obtain further information regarding colonization. The AHSWKP25 genome contained seven circular plasmids carrying multiple mobile elements and acquired antimicrobial resistance genes, reflecting the high plasticity of the *K. pneumoniae* genome. In marked contrast to *bla*_NDM–1_ and *bla*_KPC–2_ co-producing *K. pneumoniae* strains previously isolated from other parts of China, AHSWKP25 was also resistant to fosfomycin and tigecycline (Figure 2). As a common tetracycline-resistance determinant of Gram-negative bacteria, *tet(A)* can increase the efflux of tetracyclines by activating the expression of the major facilitator superfamily (MFS) efflux pumps (Nguyen et al., 2014). Tigecycline belongs to the glycylcycline family of antibiotics and has a much stronger antimicrobial activity than both tetracycline and minocycline. *tet(A)* mutants are associated with reduced bacterial susceptibility to tigecycline (Linkevicius et al., 2016). Mutated *tet(A)* can act synergistically with defective *ramR* to significantly increase the level of tigecycline resistance in *K. pneumoniae* strains (Chiu et al., 2017). Because the patient in this study had previously received tigecycline therapy, we speculated that the selection pressure exerted by tigecycline might have promoted the formation of *tet(A)* mutants (Xu et al., 2021). Furthermore, overexpression of *acrAB* and *ramA* is another common mechanism of tigecycline resistance in Enterobacteriaceae (Zhong et al., 2014; Osei Sekyere et al., 2016). In this study, we determined the role of efflux pumps in the tigecycline resistance of AHSWKP25 using the efflux pump inhibition test and RT-PCR (Figure 4). the transcriptional regulation of *acrAB* and *ramA* was closely related to the mutation in *ramR* (Abouzeed et al., 2008). Previous studies showed that the A19V substitution mutation in the *ramR* gene was more common in tigecycline and carbapenem-resistant *K. pneumoniae* (Sheng et al., 2014; Chiu et al., 2017). However, we did not find the A19V mutation in the *ramR* of AHSWKP25, rather, we did identify a D152Y substitution mutation that was previously identified in a tigecycline-non-susceptible *K. pneumoniae* strain that did not carry *tet(A)* mutant (MIC: 4 μg/ml) (Cheng et al., 2020). Thus, we considered that the tigecycline resistance of AHSWKP25 could be manifested by the combination of the *tet(A)* variant and the *ramR* mutation. *rarA* encodes an AraC-type regulator that can also independently activate the AcrAB and OqxAB efflux pumps, endowing *K. pneumoniae* with a multidrug resistance phenotype and tigecycline resistance (Veleba et al., 2012; De Majumdar et al., 2013).

Of the three antibiotic resistance plasmids carried by AHSWKP25, only the X3 plasmid pSW25NDM1 containing the *bla*_NDM–1_ gene was shown to be horizontally transferred in the present conjugation experiments. The horizontal transfer of plasmids is often more restricted in stains carrying multiple plasmids compared to strains with only single plasmid. Distorting interactions, which can affect the horizontal transfer efficiency, have previously been observed between three plasmids carried by the same host, (Gama et al., 2017a). In addition to being inhibited by fertility inhibition systems (FIN), the co-transfer of multiple plasmids is also limited in plasmids with low conjugation rates (Gama et al., 2017b). Additionally, the horizontal transfer of these resistant plasmids may result in huge fitness costs to the recipient, thereby disturbing conjugation (San Millan and MacLean, 2017). Furthermore, the host ranges of plasmids pSW25KPC2, pSW25HRG, and pSW25tet_A could be relatively narrow (Figure 3). Therefore, when assessing the horizontal transfer capacity of multiple plasmids carried by the same host, the influence of several factors must be considered. Sequence alignment showed that although the backbones of pSW25KPC2 and pSW25NDM1 were homologous to those of other plasmids isolated from Enterobacteriaceae, the plasmids acquired other genes by mobile elements, demonstrating the evolutionary potential of these antibiotic resistance plasmids (Figure 3). Because IS*CR1* can mobilize its adjacent sequences through rolling circle transposition, we speculated that the *ISCR1* element was involved in the capture of multiple antimicrobial resistance genes, including *ARR-3-dfrA27-aadA16-qacE*Δ*1-sul1* on pSW25KPC2 (Figure 3A; Cheng et al., 2016). Transposition events mediated by transposon Tn*4401* were the predominant reason for the rapid spread of the *bla*_KPC_ genes to different plasmids (Naas et al., 2008). While we did not identify the Tn*4401* transposon and its variants in pSW25KPC2, the region on pSW25KPC2 encoding *bla*_KPC–2_ was homologous with other plasmids carrying *bla*_KPC–2_. In addition, the backbone of pSW25KPC2 was highly similar to other plasmids without *bla*_KPC–2_, suggesting that the acquisition of *bla*_KPC–2_ by pSW25KPC2 can be enabled by homologous recombination between plasmids (Figures 3A, 4). Tn*As1*, Tn*3*, and IS*4321* may play a significant role in this process. Notably, our results revealed that pSW25NDM1 carrying *bla*_NDM–1_ was capable of horizontal transfer to the recipient strain *E. coli* J53 and a variety of other wide hosts (Figure 3D), highlighting the flexibility of plasmids carrying *bla*_NDM–1_ in the formation of *bla*_NDM–1_ and *bla*_KPC–2_ co-producing strains (Gao et al., 2020).

The virulence phenotype of AHSWKP25 was also a cause for concern (Figure 1). Our results also supported the observation that *K. pneumoniae* strains did not carry *rmpA*/*rmpA2*, *iucA*, and capsular serotype other than K1, K2, K5, K20, K54, and K57 could be had a hypervirulent phenotype (Supplementary Table 4). However, the accuracy of distinguishing cKp and hvKp strains based on the *G. mellonella* infection model was insufficient compared to the murine model (Russo and MacDonald, 2020). Therefore, the lack of a mammalian infection model for evaluating the pathogenicity of AHSWKP25 was a limitation of this study. In hvkp strains, the emergence of tigecycline resistance mediated predominantly by *ramR* mutations was accompanied by decreased mucoviscosity and serum resistance (Park et al., 2020; Di Pilato et al., 2022). This phenomenon can be explained by the fitness cost, meaning, in the absence of antibiotic selection, resistance mutations could disturb the physiological function of bacteria, resulting in reduced pathogenicity, growth rate, and competitiveness compared to susceptive strains accordingly (Andersson and Hughes, 2010). However, several recent studies have revealed that genes and their regulators responsible for capsule biosynthesis play a dominant role in the adaptive evolution of *K. pneumoniae*. Disruption to these genes (*rsAB*, *lon*, and *csrD*) can ultimately affect capsule production, especially if these disruptions promote capsule hyperproduction, which would lead to enhancements in the pathogenicity and serum resistance of *K. pneumoniae* (Ernst et al., 2020; Mike et al., 2021). And mutations in the *pal* are mainly associated with reduced virulence (Hsieh et al., 2013). As mentioned above, missense mutations were identified in the *rcsA*, *lon*, and *csrD* genes that regulate capsule production in AHSWKP25. In particular, the S35N missense mutation in the *rcsA* gene of the AHSWKP25 genome was previously identified in a hypervirulent and hypermucoviscous *K. pneumoniae* isolate with hypercapsule production (Morales-León et al., 2021). The hypercapsule production is not necessarily associated with hypermucoviscosity (Mike et al., 2021). Therefore, the hypervirulent phenotype of AHSWKP25 was more likely to be associated with hypercapsule production through the regulators of capsule production mutations, but the exact mechanism remains to be further studied. The Type 3 fimbriae-encoding genes *mrkABCDFHIJ* and the Type I fimbriae-encoding gene *fimABCEFGHIK* are common in ckp and hvkp strains and can promote bacterial adhesion during pathogenic infection and biofilm formation (Di Martino et al., 2003). Among the siderophores carried by AHSWKP25, enterobactin is produced in both cKp and hvKp, whereas salmonellin is found more commonly in hvKp (El Fertas-Aissani et al., 2013; Lee I. R. et al., 2016). Furthermore, the heavy metal resistance genes carried by pSW25HRG were highly homologous to the gene carried by the virulence plasmid pK2044 and might be associated with the homologous recombination in these variable regions carrying heavy metal resistance genes of virulence plasmids. Although previous studies of *S. aureus* and *A. baumannii* demonstrated a relationship between these heavy metal resistance genes and pathogenicity, their contribution to the virulence of *K. pneumoniae* strains is still unclear (Alquethamy et al., 2019; Lawal et al., 2021). Overall, we determined that virulence of AHSWKP25 differ from those of common hypervirulent strains, and our results suggested that the emergence of hvkp strains might not depend exclusively on the genetic backgrounds and that point mutations in chromosomal loci also contributed to the development of the hypervirulent phenotype (Ernst et al., 2020).

There were several other limitations of this study that must be addressed in subsequent studies. First, although we reported NDM-1 and KPC-2 co-producing *K. pneumoniae* in Southwest China for the first time, the local prevalence of such microorganisms requires further clarification. Second, as mentioned above, mammalian models are still necessary to further elucidate the virulence behavior of AHSWKP25 in the future. Third, we used only *E. coli* J53 as the recipient in the conjugation experiments, in subsequent studies, we will use *K. pneumoniae* stains as recipients to assess the horizontal transferability of these plasmids.

## Conclusion

In conclusion, this study was the first to describe and sequence a hypervirulent tigecycline-resistant and serum-resistant *K. pneumoniae* strain containing both *bla*_NDM–1_ and *bla*_KPC–2_ in Southwestern China. The resistance phenotypes displayed by AHSWKP25 suggested that *bla*_NDM–1_ and *bla*_KPC–2_ co-producing *K. pneumoniae* strains can potentially develop further antimicrobial resistance. Notably, we identified missense mutations in the genes associated with hypercapsule production in AHSWKP25, which will provide valuable information for illustrating the formation mechanism of hypervirulent *K. pneumoniae*. Importantly, strict monitoring measures must be taken to prevent the spread of these superbugs.

## Data Availability Statement

The datasets presented in this study can be found in online repositories. The names of the repository/repositories and accession number(s) can be found below: NCBI GenBank, CP091048-CP091055 (*K. pneumoniae* AHSWKP25).

## Ethics Statement

The study protocol was approved by the Institutional Review Board of the Affiliated Hospital of Southwest Medical University (Project No. KY2020043).

## Author Contributions

JH, BZ, JD, and JL isolated AHSWKP25 and designed the study and experiments. JH, JD, and YW performed the assays. JH, BZ, and XX analyzed the data. JH and BZ drafted and revised the manuscript. All authors contributed to manuscript revision, read, and approved the submitted version.

## Conflict of Interest

The authors declare that the research was conducted in the absence of any commercial or financial relationships that could be construed as a potential conflict of interest.

## Publisher’s Note

All claims expressed in this article are solely those of the authors and do not necessarily represent those of their affiliated organizations, or those of the publisher, the editors and the reviewers. Any product that may be evaluated in this article, or claim that may be made by its manufacturer, is not guaranteed or endorsed by the publisher.

## Footnotes

1
https://www.fda.gov

2
http://www.genomicepidemiology.org/

3
http://www.mgc.ac.cn/VFs/

4
https://www-is.biotoul.fr/blast.php

5
https://blast.ncbi.nlm.nih.gov/Blast.cgi

6
https://sourceforge.net/projects/mauve/

7
http://www.phyloviz.net/

8
https://itol.embl.de/

9
http://bigsdb.pasteur.fr
