# Supplementary material for: Emergence of a Hypervirulent Tigecycline-Resistant Klebsiella pneumoniae Strain Co-producing blaNDM–1 and blaKPC–2 With an Uncommon Sequence Type ST464 in Southwestern China
Source: Front Microbiol. 2022 Apr 29;13:868705. doi: 10.3389/fmicb.2022.868705 (PMC9100695; doi:10.3389/fmicb.2022.868705)
Supplement: Supplementary file 1 [file Data_Sheet_1.docx]

Supplementary Material

# Supplementary Figures and Tables

## Supplementary Figures


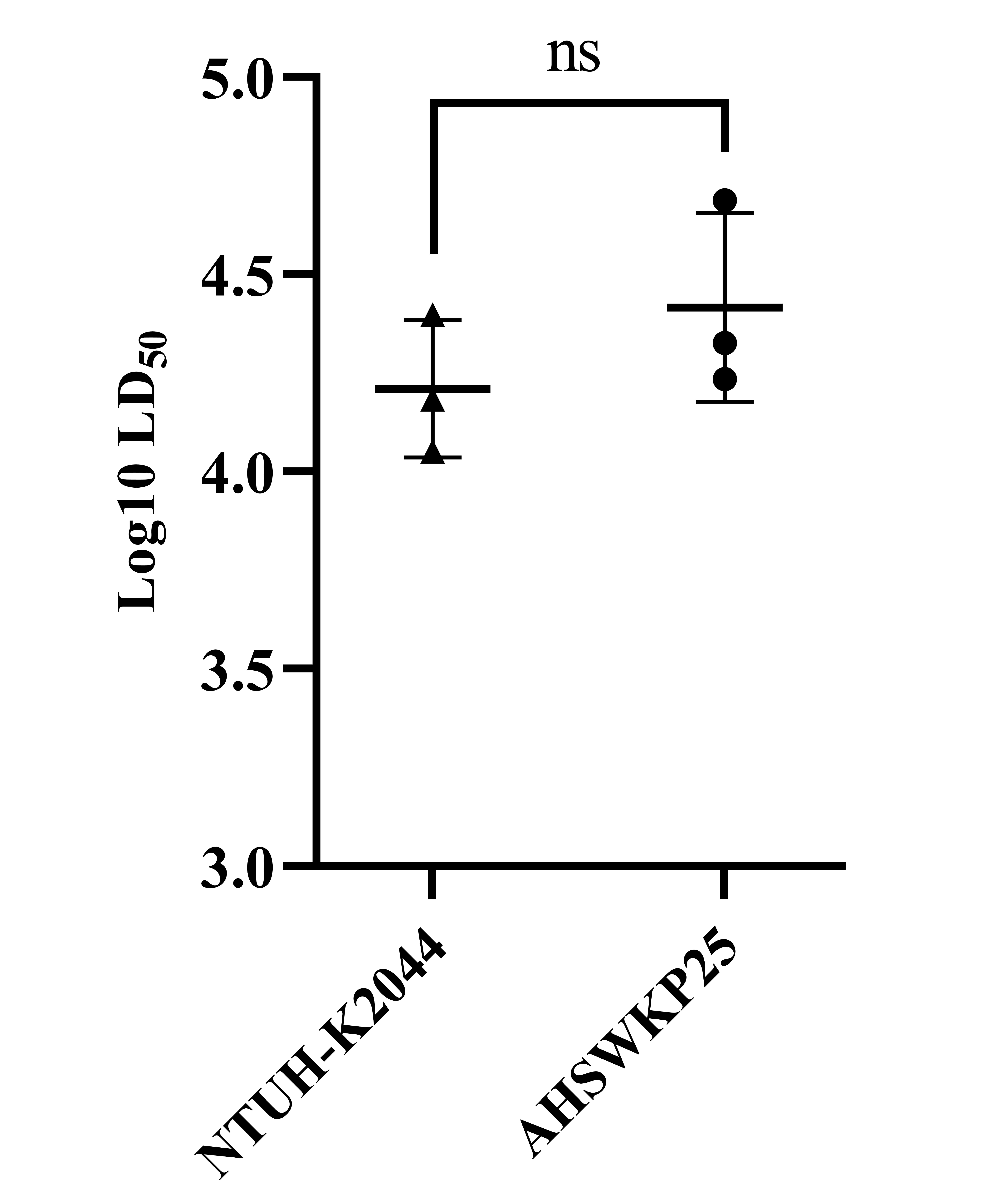


**Supplementary Figure 1.** Log10 LD_50_ (CFU) of *K. pneumoniae* AHSWKP25 and NTUH-K2044 in the *G. mellonella* infection model. The error bar represents the standard deviation (SD) of the mean. ns, no significance by Student’s t-test.


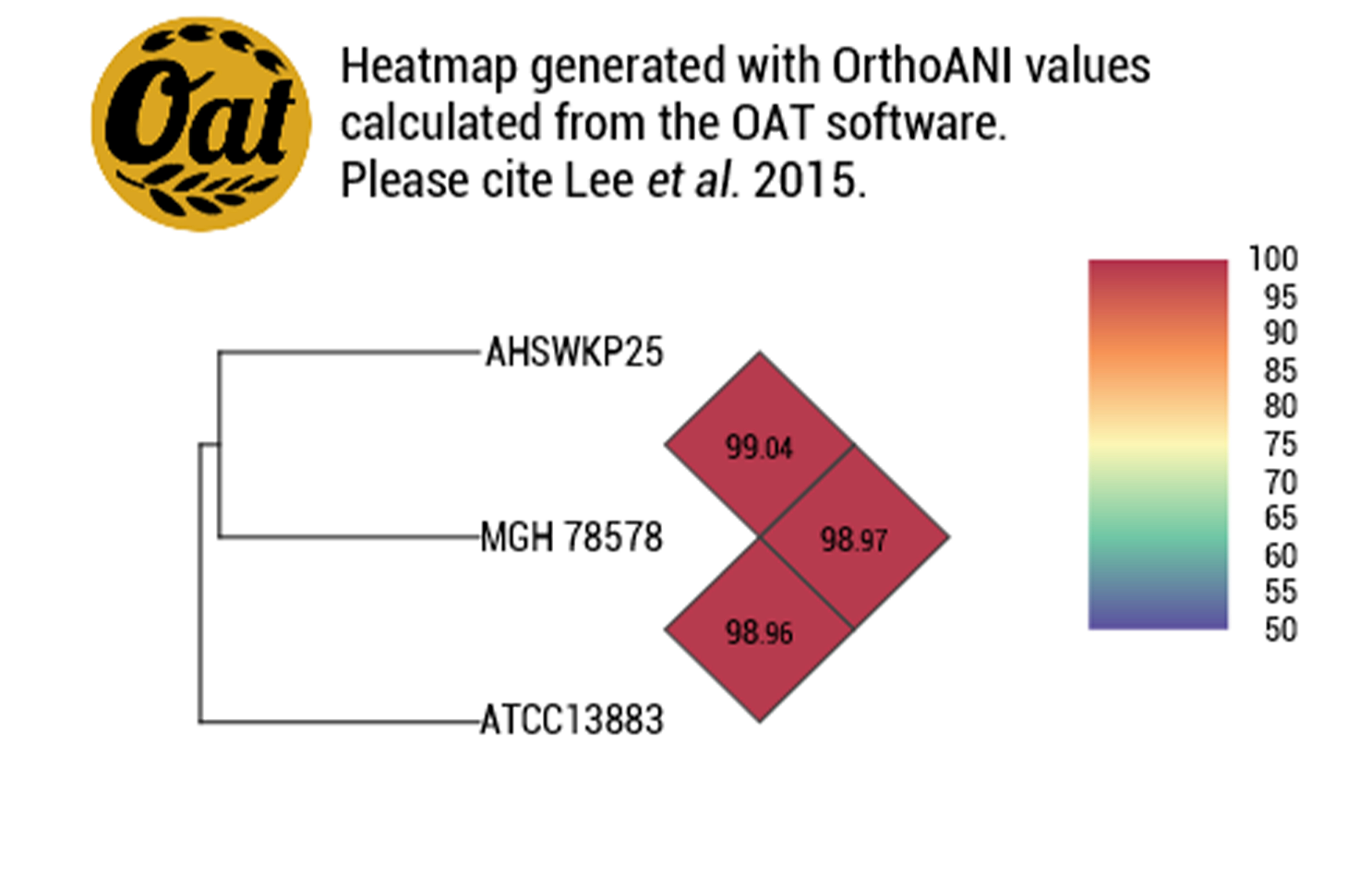


**Supplementary Figure 2**. OrthoANI values between AHSWKP25, *K. pneumoniae* ATCC13883 and MGH78578.


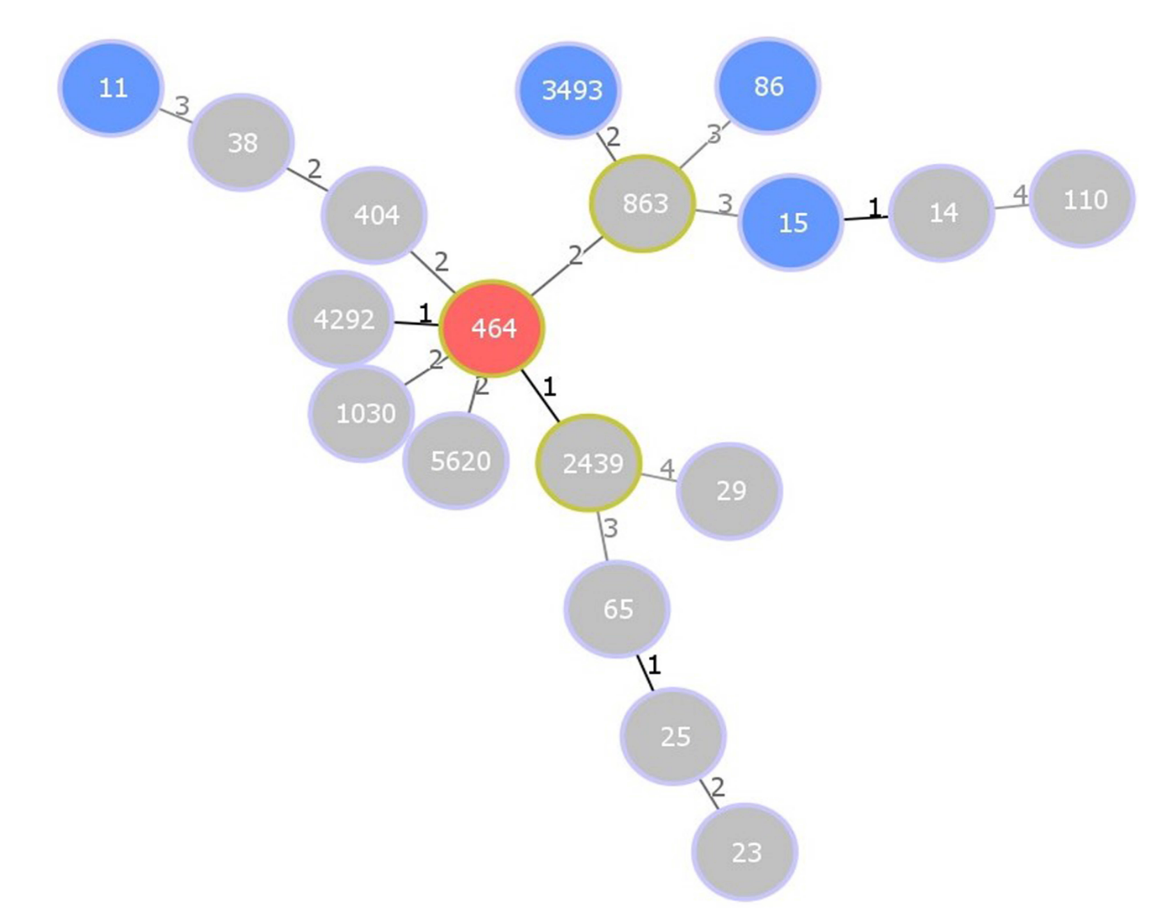


**Supplementary Figure 3.** The phylogeny among sequence types (STs) of NDM-1 and KPC-2 co-producing *K.pneumoniae* strains isolated from China analyzing by PHYLOViZ. Red: this study (ST464); Blue: strains isolated from other parts of China.


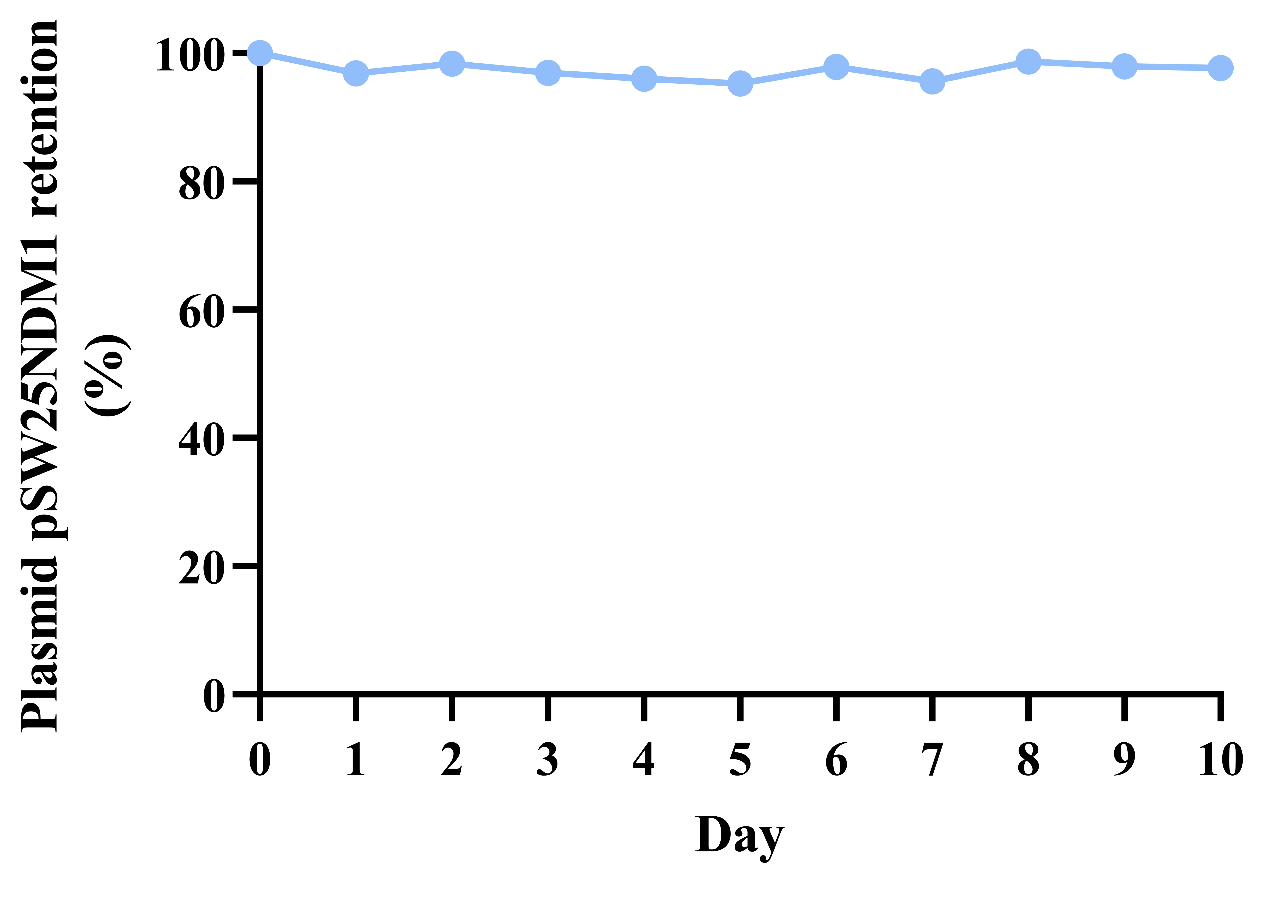


**Supplementary Figure 4.** Stability of pSW25NDM1 in *E. coli* J53 strains over 10 days.

## Supplementary Tables

**Supplementary Table** **1.** PCR and RT-PCR primers used in this study.

| PCR | **Genes** | **Forward (5’-3’)** | **Reverse (5’-3’)** | **References** |
| --- | --- | --- | --- | --- |
|  | *bla*_KPC_ | CATTCAAGGGCTTTCTTGCTGC | ACGACGGCATAGTCATTTGC | (Dallenne et al., 2010) |
|  | *bla*_NDM_ | CGGAATGGCTCATCACGATC | GGTTTGGCGATCTGGTTTTC | (Krishnaraju et al., 2015) |
| RT-PCR | *acrA* | CAGCAGGGCGTCAGGGTTAATC | GATCAGACCACCGGCTCAATCAC | This study |
|  | *acrB* | TGTCCGAAGCGAAATACCGTATGC | CAGACCGTTGTAGCGTTCCAGAC |  |
|  | *marA* | CGGGATTCGCCAGGTACATTCG | CTATGGTTTCGAGTCGCAGCAGAC |  |
|  | *marR* | GGCAGGACCTTCTTGAGCAGTTG | CGAGCAGTGTCATCAGGTTGTGG |  |
|  | *ramA* | TGCCTCAAGTATGGCTTCGATTCG | GACTGTGGTTCTCTTTGCGGTAGG |  |
|  | *rarA* | GCAACAGAGCGGCTGATACTCC | CCGATCCTGAACGTGGCGATTG |  |
|  | *rpoB* (Internal reference) | GCGTCTGTCTCTTGGCGATCTG | AGCGGGTTGTTCTGGTCCATAAAC |  |

Dallenne, C., Da Costa, A., Decré, D., Favier, C., and Arlet, G. (2010). Development of a set of multiplex PCR assays for the detection of genes encoding important beta-lactamases in Enterobacteriaceae. *J. Antimicrob. Chemother.* 65(3)**,** 490-495. doi: 10.1093/jac/dkp498

Krishnaraju, M., Kamatchi, C., Jha, A.K., Devasena, N., Vennila, R., Sumathi, G., et al. (2015). Complete sequencing of an IncX3 plasmid carrying blaNDM-5 allele reveals an early stage in the dissemination of the blaNDM gene. *Indian J. Med. Microbiol.* 33(1)**,** 30-38. doi: 10.4103/0255-0857.148373

**Supplementary Table 2.** Basic information on genomes (sequences) obtained from the NCBI database.

| **Name** | **Accession number** | **Host/Species** |
| --- | --- | --- |
| pK55602_2 | CP042976.1 | *K. pneumoniae* |
| pKPC2_095132 | CP028389.3 | *K. pneumoniae* |
| pKP04VIM | KU318421.1 | *K. pneumoniae* |
| pYNKP001-dfrA | KY270853.1 | *R. ornithinolytica* |
| pRJA166a | CP019048.1 | *K. pneumoniae* |
| pK2044 | NC_006625.1 | *K. pneumoniae* |
| pCAV1042-183 | CP018670.1 | *K. pneumoniae* |
| pRGT34-2-122k | CP075310.1 | *K. pneumoniae* |
| tig00000195 | CP021946.1 | *K. pneumoniae* |
| pECL-14-60-NDM-1 | MN061454.1 | *E. cloacae* |
| pLEC-b38d | CP026168.1 | *Leclercia* sp. |
| pNDM1_005008 | CP058664.1 | *E. coli* |
| pNDM-XZA88 | CP076461.1 | *K. pneumoniae* |
| Ror-30818cz | MG252893.1 | *R. ornithinolytica* |
| C2315 | PRJNA534178 | *K. pneumoniae* |
| C2343 |  | *K. pneumoniae* |
| C2414 |  | *K. pneumoniae* |
| C2601 |  | *K. pneumoniae* |
| C2660 |  | *K. pneumoniae* |
| C2972 |  | *K. pneumoniae* |
| C2974 |  | *K. pneumoniae* |
| CRKP380 | SAMN13735565 | *K. pneumoniae* |
| NUHL30457 | SAMN08455837 | *K. pneumoniae* |

**Supplementary Table 3.** Serum killing assay results (log_10_-transformed values, mean ± SEM)

| **Strains** | **0h** | **1h** | **2h** | **3h** | **Grade** |
| --- | --- | --- | --- | --- | --- |
| ATCC700603 | 4.607±0.04 | 3.688±0.04 | 2.466±0.28 | 1.163±0.23 | highly sensitive |
| AHSWKP25 | 4.548±0.07 | 4.635±0.05 | 4.621±0.06 | 3.87±0.37 | resistant |
| NTUH-K2044 | 4.597±0.10 | 4.627±0.10 | 4.631±0.10 | 4.074±0.40 | resistant |
